# Supplementary material for: Semicarbazide-sensitive amine oxidase inhibition ameliorates albuminuria and glomerulosclerosis but does not improve tubulointerstitial fibrosis in diabetic nephropathy
Source: PLoS One. 2020 Jun 18;15(6):e0234617. doi: 10.1371/journal.pone.0234617 (PMC7302447; doi:10.1371/journal.pone.0234617)
Supplement: Attachment — Submitted filename: response to reviewers. CPdocx.docx [file pone.0234617.s001.docx]

6^th^ April 2020

Jaap A. Joles, DVM, PhD
Academic Editor
PLOS ONE

Dear Dr Joles and reviewers,

Re: PONE-D-19-34728 Semicarbazide-sensitive amine oxidase (SSAO) inhibition ameliorates glomerulosclerosis but does not improve tubulointerstitial fibrosis in diabetic nephropathy

Thank you for the opportunity to make changes to our manuscript. We hope it meets your requirements.

Kind regards,

A/Prof. Sonia Saad: 
on behalf of: May YW Wong, Muh Geot Wong, Stefanie Stangenberg, Wolfgang Jarolimek, Heidi Campos Schilter, Amgad Zaky, Anthony J. Gill, Carol Pollock 

1. Please ensure that your manuscript meets PLOS ONE's style requirements, including those for file naming. The PLOS ONE style templates can be found at <http://www.journals.plos.org/plosone/s/file?id=wjVg/PLOSOne_formatting_sample_main_body.pdf> and <http://www.journals.plos.org/plosone/s/file?id=ba62/PLOSOne_formatting_sample_title_authors_affiliations.pdf>

We have made sure that PLOS ONE style requirements have been used.

2. We noticed you have some minor occurrence(s) of overlapping text with the following previous publication(s), which needs to be addressed: In your revision ensure you cite all your sources (including your own works), and quote or rephrase any duplicated text outside the Methods section. Further consideration is dependent on these concerns being addressed.

This has been closely checked and overlapping text and content changed. Several articles referenced were written by the same primary author – these areas have been changed and/or referenced.

3. As part of your revision, please complete and submit a copy of the ARRIVE Guidelines checklist, a document that aims to improve experimental reporting and reproducibility of animal studies for purposes of post-publication data analysis and reproducibility: <https://www.nc3rs.org.uk/arrive-guidelines>. Please include your completed checklist as a Supporting Information file. Note that if your paper is accepted for publication, this checklist will be published as part of your article.

This document is now filled out and attached to submission

4. In your Methods section, please give the sources of all reagents used in your study, including telmisartan.

These have been added for Telmisartan (Boehringer-Ingelheim, Germany), PXS-4728A (Pharmaxis Pty Ltd, Frenchs Forest, Australia) and ^14^C- benzylamine/benzylamine solution (200M, PerkinElmer, Meridin, CT)

5. We note that one or more of the authors are employed by a commercial company: Pharmaxis.

1. Please provide an amended Funding Statement declaring this commercial affiliation, as well as a statement regarding the Role of Funders in your study. If the funding organization did not play a role in the study design, data collection and analysis, decision to publish, or preparation of the manuscript and only provided financial support in the form of authors' salaries and/or research materials, please review your statements relating to the author contributions, and ensure you have specifically and accurately indicated the role(s) that these authors had in your study. You can update author roles in the Author Contributions section of the online submission form. Please also include the following statement within your amended Funding Statement. “The funder provided support in the form of salaries for authors [insert relevant initials], but did not have any additional role in the study design, data collection and analysis, decision to publish, or preparation of the manuscript. The specific roles of these authors are articulated in the ‘author contributions’ section.” If your commercial affiliation did play a role in your study, please state and explain this role within your updated Funding Statement.

Pharmaxis (http://www.pharmaxis.com.au) supported the study in addition to a successful peer reviewed application to the Australian Research Council. WJ provided critical scientific review in the preparation of the manuscript but the content of the manuscript was at all times decided by the academic investigators. They approved the study design but were not directly involved. They were not directly involved with data collection or analysis. HS provided support with the SSAO activity assay. Both WJ and HS reviewed the manuscript prior to publication but were not involved in the decision to publish nor were they involved in the preparation of the manuscript.

Acknowledgements

The study was a collaborative work with Pharmaxis Ltd. funded by the Australia Research Council (ARC) Linkage Grant

Author Contributions

M.Y.W., S.Sa. and M.G.W. conceived and designed the experiments. M.Y.W and S.St. performed the experiments. M.Y.W. and M.G.W. analysed the data. A.Z. and A.G. contributed to the histological preparation of the slides. M.Y.W wrote the paper. H.S, W.J, S.Sa., S.St, M.G.W. and C.A.P. reviewed the paper.

Competing Interests

W.J and H.S. are employees of Pharmaxis Ltd.

2. Please also provide an updated Competing Interests Statement declaring this commercial affiliation along with any other relevant declarations relating to employment, consultancy, patents, products in development, or marketed products, etc. Within your Competing Interests Statement, please confirm that this commercial affiliation does not alter your adherence to all PLOS ONE policies on sharing data and materials by including the following statement: "This does not alter our adherence to  PLOS ONE policies on sharing data and materials.” (as detailed online in our guide for authors <http://journals.plos.org/plosone/s/competing-interests>) . If this adherence statement is not accurate and  there are restrictions on sharing of data and/or materials, please state these. Please note that we cannot proceed with consideration of your article until this information has been declared.

Competing Interests Statement. MYW: None, MGW: None ,SS: None WJ, AZ: None, AJG: None, CP, None SSaad: None, : Employee of Pharmaxis , HS: Employee of Pharmaxis. This does not alter our adherence to  PLOS ONE policies on sharing data and materials.

**Reviewers' comments:**

**Reviewer #1:** This study is significant in establishing a valid DKD model in which the protection of SSAOi against DKD is demonstrated as is the case in the human clinical study. It also proved the amelioration of glomerulosclerosis, which could be inferred from the fact that SSAOi decreases UACR. However, the exploration of the protection mechanism of SSAOi needs some improvement. Although the authors proved that the target of SSAOi-induced protection in this model was the glomeruli, in subsequent experiments they seemed to pursue markers of tubulointerstitial fibrosis and inflammation. It is recommended that the experiments elucidating the protection mechanism of glomeruli are conducted.

Major issues:

1) The localization of SSAO in the DKD model should be elucidated. In our previous study (Tanaka S, et al. Kidney Int. 2017;92: 154-164), we demonstrated that VAP-1 was most abundant in the pericytes in the interstitium of corticomedullary junction in healthy kidneys. In the DKD model employed in this study, the localization might be different from the physiological condition.

Unfortunately, we were unable to determine specific localisation of SSAO with immunohistochemistry stains as the expression was too low. However, as shown in Figure 7, we have demonstrated that SSAO activity is increased in the diabetic kidney. We have included the following in the discussion “Although the pattern of VAP-1 expression in the kidney was not demonstrated in this study, Tanaka et al. (Kidney Int. 2017;92: 154-164), previously demonstrated that VAP-1 is mostly abundant in pericytes. It has been previously shown that the kidneys of transgenic mice over-expressing VAP-1 in their endothelium develop glomerulosclerosis (Stolen et al, The FASEB journal. 2004; 18: <https://doi.org/10.1096/fj.03-0562fje>). They also found high expression of the receptor for advanced glycosylation end products at the vascular pole of renal corpuscles from mice fed the atherogenic diet. This difference between the Tanaka study and our findings may be related to the difference in models; Tanaka used the kidneys of healthy mice whereas we used kidneys from a diabetic model (endothelial nitric oxide synthase knockout (eNOS^-/-^) mice on a C57BL/6 background).” We have updated the references accordingly.

2) ROS level and leukocyte infiltration should be evaluated specifically in the glomeruli.
The levels of CD45 (pan-leukocyte marker) and Nitrotyrosine (a marker of ROS) in the glomeruli are now shown in Fig 6B and 5B respectively. The result section and discussion sections are updated.

3) The type of leukocytes reduced in the treated diabetic kidneys should be elucidated. In our same previous study, we showed that VAP-1 inhibition significantly decreased neutrophil aggregation. I wonder if this is the case with this model, given the number of macrophages were not significantly changed.

Unfortunately we cannot address this specific question because of logistic issues. Our laboratory has been closed because of coronavirus -related quarantine procedures. We believe that this experiment is not necessary and will not change the main finding of the study. We have included this as a limitation in the discussion section. The following statement is now added “Tanaka et al. demonstrated that VAP-1 inhibition significantly decreases neutrophil aggregation in a rat model of renal ischaemia/reperfusion injury. It remains unclear whether it also inhibits leukocyte aggregation in diabetic kidney disease. However, neutrophil aggregation is not a documented feature of DKD.” The references are also updated accordingly.

4) The Discussion should be more focused on glomerulosclerosis rather than interstitial fibrosis, given that the tubulointerstitial injury was not significantly different with SSAOi treatment in this model.

We redirected the focus of discussion on glomerulosclerosis and have moved the interstitial fibrosis to the later section of the manuscript, to place more focus on the role of glomerular injury.

Minor issue:

1) Table 2 should include the information of blood pressure.

The blood pressure of animals was not assessed in this study. However, our group has extensively studied the diabetic eNOS-/- model and demonstrated that blood pressure is not changed in the diabetic animals (Gangadharan Komala M, PLoS One. 2014; 9(11)e108994, Yong R, PLoS One. 2013; 8(8): e73428); Huang C, Diabetes. 2013; 62(8) 2923-2934.) We have also recently demonstrated no effect of SSAO inhibition and telmisartan on blood pressure using a unilateral ureteric obstruction model (Wong et al, <https://doi.org/10.1152/ajprenal.00698.2013>) and found no difference between groups (control, sham, SSAO inhibitor and telmisartan). This has now been included in the limitations and references.

**Reviewer #2:** This manuscript describes positive effects of SSAO/VAP-1 inhibitor in diabetic kidney disease in a mouse model. Overall, this is a solid paper. However, its impact in clinical means is compromised as another inhibitor SSAO/VAP-1 inhibitor has shown beneficial effects in clinical trials.

Whilst there have been clinical trials including phase 2a testing for diabetic retionopathy and non alcoholic steatohepatitis, this study aims to supplement the body of knowledge by looking at mechanistic aspects. Most recently, a Phase 2 trial investigating BI 1467335 in patients with clinical evidence of NASH was terminated because of concerns regarding drug interactions for patients with NASH. Furthermore, there has not been to date clinical trials in chronic kidney disease.

Major:
All figure legends need information about the number of animals used.

Many Figures are not sharp and of poor quality. Please, replace. Figure 8 especially does not give any information, as the histology is really bad in several panels.

The number of animals has been provided in the figure legends “n=6-8”. We have made sure the image sizes now range from 6-8Megabites in size and of better quality. We removed figure 8 as recommended.

Figures need scale bars. Mentioning of the original magnification becomes irrelevant.
Scale bars have been provided in all the figures and figure legends.

There are several inconsistencies, which need to be fixed:

1. Line 72: VAP-1/SSAO is not present on leukocytes

This has been removed.

2. Line 76: VAP-1 is Vascular Adhesion Protein-1, not Vascular-antigen peptide

This has been changed.

3. Line 282: BSL should be BGL

This has been changed throughout the manuscript (line 269, 273)

4. Line 311: The authors write: the urinary albumin to creatinine ratio (ACR) was greater in untreated diabetic mice (362±85 μg/mg) compared to the control group (113±17μg/mg; P<0.01 vs. Ctrl). How and why there is albumin in the urine of healthy mice??????

Whilst this is not completely expected, we and others have reported similar levels in control mice (Gangadharan Komala M, PLoS One. 2014; 9(11)e108994 and Glataras, PLoS ONE. 2016; 11(8): e0162131). The ACR range is in microalbuminuria range and was taken at time of sacrifice. It is possible this may be age related as this is a 32 week model.

5. Line 365: SSAOi administration inhibits cortical transcription fibronectin deposition in diabetic mice but not collagen IV deposition. Remove transcription from the title, because you just analyse fibronectin staining

“Transcription” has been removed from the title

6. Line 382: Representative photomicrographs of fibronectin staining cells and histograms summarising expression. Fibronectin is an extracellular matrix protein, remove cells.

Cells has been removed to read “Representative photomicrographs of fibronectin staining and histograms summarising expression”.

7. Line 404: Nitrotyrosine is a marker of cell damage, inflammation, and nitric oxide (NO) production. This type of sentence is already in the text and does not belong to a figure legend.

This has been removed from the figure legend

8. Line 432: Macrophage cells are just macrophages

The word “cells” has been removed.

9. Line 446: The authors write: SSAO activity in the diabetic kidneys treated with telmisartan was no different compared to untreated diabetic kidneys (Fig 7). However, in the figure legend they write: Diabetes resulted in increased kidney SSAO activity which is effectively suppressed by administration of an SSAO inhibitor (PXS-4782A) and Telmisartan, as well as combination therapy. Please, correct.

We apologise for the confusion. The information in the gigure legend is accurate. The result section is now corrected accordingly.

10. Line 503: The can should be this can

This has been changed

11. Line 508 onwards: Benzylamine is an artificial substrate for SSAO that does not occur naturally. It is used in enzymatic activity measurement. Thus, it cannot contribute in this scenario, if not given exogenously. Please, modify.

The section on benzylamine has been removed

12. Line 559 onwards: Delete Nitrotyrosine paragraph – it is simply result no discussion.

The nitrotyrosine paragraph has been removed from the discussion.

13. References: please check the references – there are several inconsistences

 We have checked the references and made the following changes; ref <27> and <30> Morin et al were duplicates
